# Role of Innate Inflammation in the Regulation of Tissue Remodeling during Tooth Eruption

**DOI:** 10.3390/dj9010007

**Published:** 2021-01-12

**Authors:** Yusuke Makino, Kaoru Fujikawa, Miwako Matsuki-Fukushima, Satoshi Inoue, Masanori Nakamura

**Affiliations:** Department of Oral Anatomy and Developmental Biology, Showa University School of Dentistry, Tokyo 142-8555, Japan; sdcdrm@me.com (Y.M.); kfujikawa7@dent.showa-u.ac.jp (K.F.); mfukushima@dent.showa-u.ac.jp (M.M.-F.); s-inoue@dent.showa-u.ac.jp (S.I.)

**Keywords:** HIF-1, IL-1, KC, neutrophil, tooth eruption

## Abstract

Tooth eruption is characterized by a coordinated complex cascade of cellular and molecular events that promote tooth movement through the eruptive pathway. During tooth eruption, the stratum intermedium structurally changes to the papillary layer with tooth organ development. We previously reported intercellular adhesion molecule-1 (ICAM-1) expression on the papillary layer, which is the origin of the ICAM-1-positive junctional epithelium. ICAM-1 expression is induced by proinflammatory cytokines, including interleukin-1 and tumor necrosis factor. Inflammatory reactions induce tissue degradation. Therefore, this study aimed to examine whether inflammatory reactions are involved in tooth eruption. Reverse transcription-polymerase chain reaction (RT-PCR) analysis revealed sequential expression of hypoxia-induced factor-1α, interleukin-1β, and chemotactic factors, including keratinocyte-derived chemokine (KC) and macrophage inflammatory protein-2 (MIP-2), during tooth eruption. Consistent with the RT-PCR results, immunohistochemical analysis revealed KC and MIP-2 expression in the papillary layer cells of the enamel organ from the ameloblast maturation stage. Moreover, there was massive macrophage and neutrophil infiltration in the connective tissue between the tooth organ and oral epithelium during tooth eruption. These findings suggest that inflammatory reactions might be involved in the degradation of tissue overlying the tooth organ. Further, these reactions might be induced by hypoxia in the tissue overlying the tooth organ, which results from decreased capillaries in the tissue. Our findings indicate that bacterial infections are not associated with the eruption process. Therefore, tooth eruption might be regulated by innate inflammatory mechanisms.

## 1. Introduction

Normal organ and tissue development should involve a well-harmonized balance with the degradation of surrounding tissues [1,2,3]. During tooth eruption, there are significant tissue changes between the reduced enamel epithelium that covers the forming enamel and the oral epithelium. Eventually, the tooth erupts into the oral cavity after a direct connection between the reduced enamel epithelium and oral epithelium, as well as the subsequent epithelial cell degradation.

Tooth eruption involves a coordinated complex cascade of cellular and molecular events that promote tooth movement through the eruptive pathway. Dental follicles are crucially involved in this cascade since they produce numerous factors: tumor necrosis factor-α, transforming growth factor-β, interleukin-1 (IL-1), colony-stimulating factor-1 (CSF-1), and receptor activator of nuclear factor kappa B ligand (RANKL). TNF-α, CSF-1, and RANKL are released by dental follicle cells and stimulate the migration and differentiation of bone marrow precursor mononuclear cells into osteoclasts [4,5,6]. Moreover, mononuclear cells migrate into the overlying connective tissues of tooth organs, which are known as dental follicles, at tooth eruption onset [7,8,9,10] to form osteoclasts for resorption of alveolar bones. All these factors are induced by inflammation onset, which is often induced by infection. Further, most matrix proteinases, including matrix metalloproteinases (MMPs), are induced by inflammatory cytokines.

Extracellular matrix remodeling is a prominent feature of various biological processes, which range from wound healing and tissue development to tumor invasion and chronic inflammatory disorders. These processes are maintained by sequential regulation of inflammatory cytokine and chemokine expression in the local region without stimulation by infection.

There have been recent reports on the association of noninfectious innate inflammation with several diseases, including rheumatoid arthritis and lung diseases [11,12]. Innate inflammation is induced by noninfectious stimuli, including degradative products during cell death [13].

We previously demonstrated that inflammatory cells regulate organ development and homeostasis [14,15,16]. Further, we observed intercellular adhesion molecule-1 (ICAM-1) expression in the enamel organ during development [17,18]. ICAM-1 expression is regulated by inflammatory cytokines [19,20]. These findings strongly indicated that the inflammatory system also regulates noninfectious tooth eruption. During tooth eruption, there is a decrease in the number of blood vessels [21]. Hypoxia-induced factor-1 (HIF-1) is the central regulator for detecting cellular oxygen levels [22] and is among the essential inflammation regulators [23,24].

This study aimed to analyze the expression of HIF-1, as well as inflammatory cytokines and chemokines, and to examine macrophage and neutrophil migration into the tissues overlying the tooth organ.

## 2. Materials and Methods

### 2.1. Animals

The experimental protocols were reviewed and approved by the Animal Care Committee of Showa University (Shinagawa-ku, Tokyo, Japan). We purchased 10 pregnant BALB/c mice at 16-day gestation (E16) from Sankyo Laboratory Service Corporation (Tokyo, Japan) and maintained them under routine conditions at the Laboratory Animal Center of Showa University. We examined the mandibles from the mice at 7 days to 21 days postnatal (dPN). Twenty-one mice were used for histological examination (n = 7, for 7, 14, 19 dPN) and 18 mice were used for analyzing relative gene expression levels (n = 9, for 10, 14 dPN).

### 2.2. Tissue Preparation

The mice were anesthetized with isoflurane and euthanized with cervical dislocation. Regarding histological and immunohistochemical studies, the mandibles were dissected from the mice and fixed using 4% paraformaldehyde for 6 h at 4 °C. After decalcification using 10% ethylenediaminetetraacetate for 2 weeks at 4 °C, the specimens were embedded and frozen in the optimal cutting temperature compound (Sakura, Torrance, CA, USA). Sagittal serial frozen sections that were parallel to the long axis of the first molars were cut (10-μm thick) and placed on glass slides. Every third section underwent hematoxylin and eosin staining and was evaluated through light microscopy to determine the suitable tooth organ region for assessment. The remaining sections were processed for immunohistochemical evaluation.

### 2.3. Immunohistochemistry

The following primary antibodies were diluted 1:100 and used: rat anti-mouse Gr-1 (553126, BD Pharmingen, Tokyo, Japan) and F4/80 (565409, BD Pharmingen) monoclonal antibodies, as well as goat anti-mouse macrophage inflammatory protein-2 (MIP-2/CXCL2) (AF-452-NA, R&D Systems, Minneapolis, MN, USA) and anti-mouse keratinocyte-derived chemokine (KC/CXCL1) polyclonal antibodies (AF-453-NA, R&D Systems). Rat anti-mouse ICAM-1 monoclonal antibody was used to identify ICAM-1-expressing cells (550287, BD Pharmingen). After washing serial tissue sections in phosphate-buffered saline (PBS), they were immersed in PBS containing 1% H_2_O_2_ for 30 min to block endogenous peroxidase activity. Subsequently, the sections were incubated with normal goat or horse serum for 30 min at room temperature. The sections were incubated with primary antibodies for 24 h at 4 °C and, subsequently, washed thoroughly using PBS. The sections were incubated with biotin-conjugated goat anti-rat IgG antibody (BA-9400, Vector Laboratories, Burlingame, CA, USA) or rabbit anti-goat IgG antibody (BA-1000, Vector Laboratories) for 60 min; subsequently, they were incubated with avidin-biotin-peroxidase complex using a Vectastain Elite ABC Kit (PK-6100, Vector Laboratories) for 30 min. Color development was performed using 3, 3′-diaminobenzidine tetrahydrochloride in TRIS buffer plus hydrogen peroxide (54-10-00, DAB Reagent set, KPL, Gaithersburg, MD, USA). The sections were counterstained with hematoxylin or methyl green. Normal rat or goat serum was used as a negative control in place of the primary and secondary antibodies.

### 2.4. Quantitative Real-Time PCR Analysis

The oral mucosa was microdissected from mice at P10 and P14 (nine mice, respectively). Subsequently, total RNA was extracted using TRIzol (Thermo Fisher Scientific, Tokyo, Japan). Complementary DNA (cDNA) was generated using the Primer Script RT Reagent Kit and gDNA Eraser (Takara Bio Inc., Shiga, Japan). Real-time PCR was performed using the Light Cycler 96 System (Roche Diagnostics, Tokyo, Japan) and TaqMan™ Fast Advanced Master Mix (Thermo Fisher Scientific, Tokyo, Japan). We amplified cDNA using the TaqMan Gene Expression assay, Mm01336189_m1 (IL-1β), Mm00468869_m1 (HIF-1α), Mm00436450_ml (MIP-2), Mm00433859_m1 (KC), and Mm99999915_g1 (GAPDH). GAPDH mRNA levels were quantified as internal controls. The PCR conditions were as follows: polymerase activation at 95 °C for 20 s, followed by 40 denaturation cycles at 95 °C for 3 s and annealing/extension at 60 °C for 30 s. Relative expression levels were calculated using the ∆(∆Ct) method. Nine different samples for each day were used, and all examinations were performed in four times technical duplicates per subject.

### 2.5. Statistical Analysis

The results are expressed as mean ± standard deviation. Group comparisons were performed using a one-way analysis of variance (ANOVA), and statistical significance was determined using Tukey’s comparison test. Statistical significance was set at *p*-values < 0.05.

## 3. Results

### 3.1. ICAM-1 Expression during Tooth Eruption

**Figure 1:**
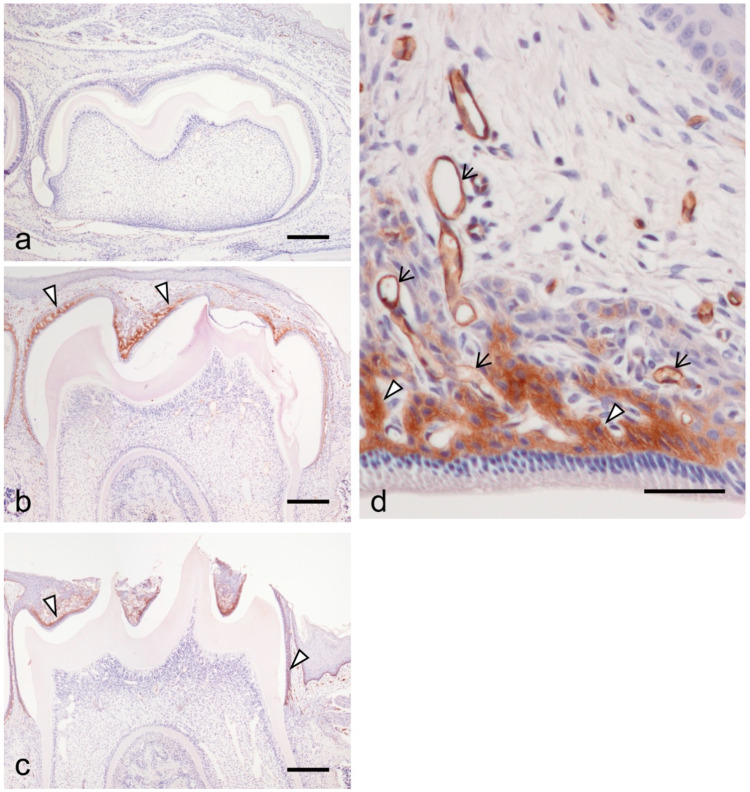
The expression of intercellular adhesion molecule-1 (ICAM-1) during the tooth organ development at 7 days postnatal (dPN) (**a**), 14 dpN (**b**), and 19 PN (**c**,**d**). (**a**) No clear expression of ICAM-1 was detected in the tooth organ. (**b**) ICAM-1 was detected at the papillary layer cells (arrowheads). (**c**) Strong reactions of ICAM-1 expression were also detected in the papillary layer (arrowheads). (**d**) Higher magnification of the enamel organ at 19 dPN. ICAM-1 expressed in blood vessels (arrows) in addition to the papillary layer (arrowheads). Bars = 200 μm in (**a**–**c**); 50 μm in (**d**).

At 7 dPN, the inner enamel epithelium of the first mandibular molar tooth organ differentiated into the secretory stage of ameloblasts and secreted enamel matrix on the dentin surface (Figure 1a). The stratum intermedium was formed on secretory ameloblasts. None of the cells of the enamel organ expressed ICAM-1 (Figure 1a). At 14 dPN, the stratum intermedium structurally changed to the papillary layer concomitantly with ameloblast differentiation from the secretory-maturation stage. There was critical ICAM-1 expression on the papillary layer cells (Figure 1b, arrowheads). At 19 dPN, the crown tips were exposed to the oral cavity. There was strong ICAM-1 expression in the papillary layer (Figure 1c,d, arrowheads) and blood vessels surrounding the enamel organ (Figure 1d, arrows).

### 3.2. Chemokine and Cytokine Expression during Tooth Eruption

We observed remarkable modification of the tissue overlying the first mandibular molar tooth organ. Consequently, quantitative real-time PCR was used to compare inflammatory chemokine and cytokine expression at this interval.

**Figure 2:**
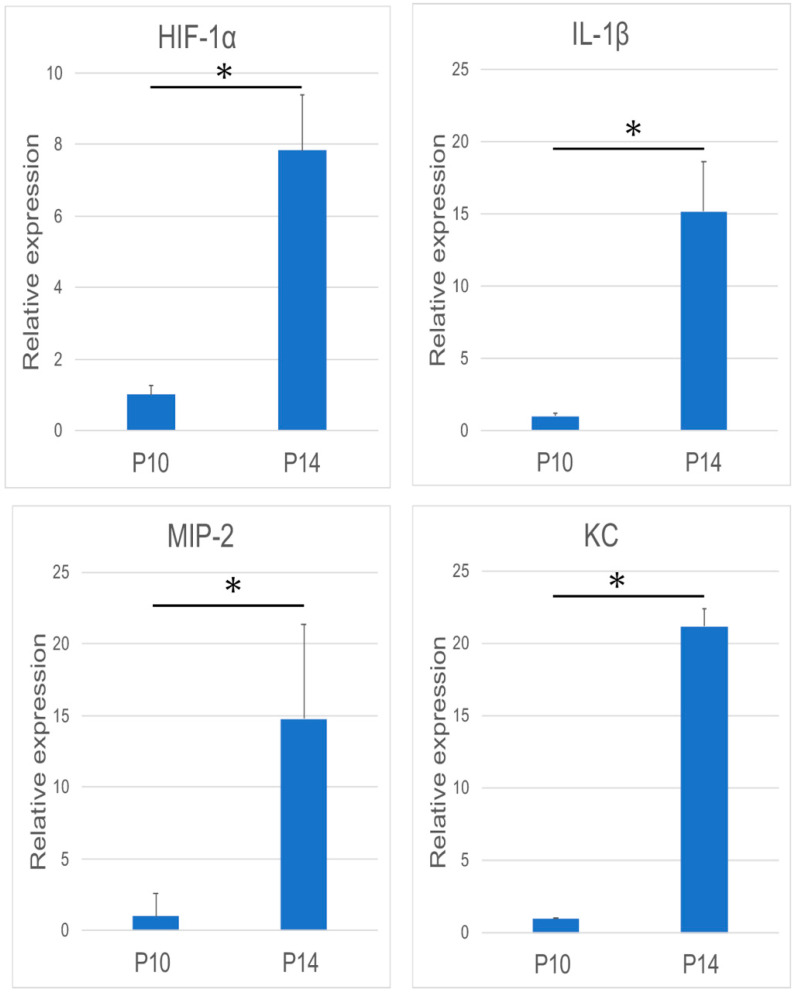
Analysis of the mRNA expression of Mm00468869_m1 (HIF-1α), Mm01336189_m1 (IL-1β), macrophage inflammatory protein-2 (MIP-2), and keratinocyte-derived chemokine (KC) using reverse transcription-polymerase chain reaction (RT-PCR) (n = 9 in each group). In general, the mRNA expression of these four molecules was significantly increased during the tooth eruption. (* *p* < 0.05).

**Figure 3:**
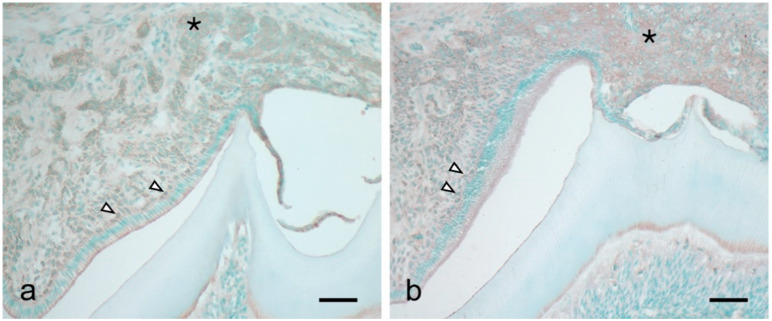
Immunohistochemical detection of KC (**a**) and MIP-2 (**b**) in the tooth organ at 14 dPN. Both chemokines were expressed in the papillary layer (arrowheads) and adjacent odontogenic epithelial cells over the tooth organs (asterisk). Bars = 50 μm.

There was significant augmentation of HIF-1α, IL-1β, MIP-2, and KC expression at 14 dPN (Figure 2). Immunohistochemical studies revealed KC and MIP-2 expression in the papillary layer and adjacent odontogenic epithelial cells over the tooth organs (Figure 3a,b, arrowheads and asterisk).

### 3.3. Migration of Macrophages and Neutrophils during Tooth Eruption

**Figure 4:**
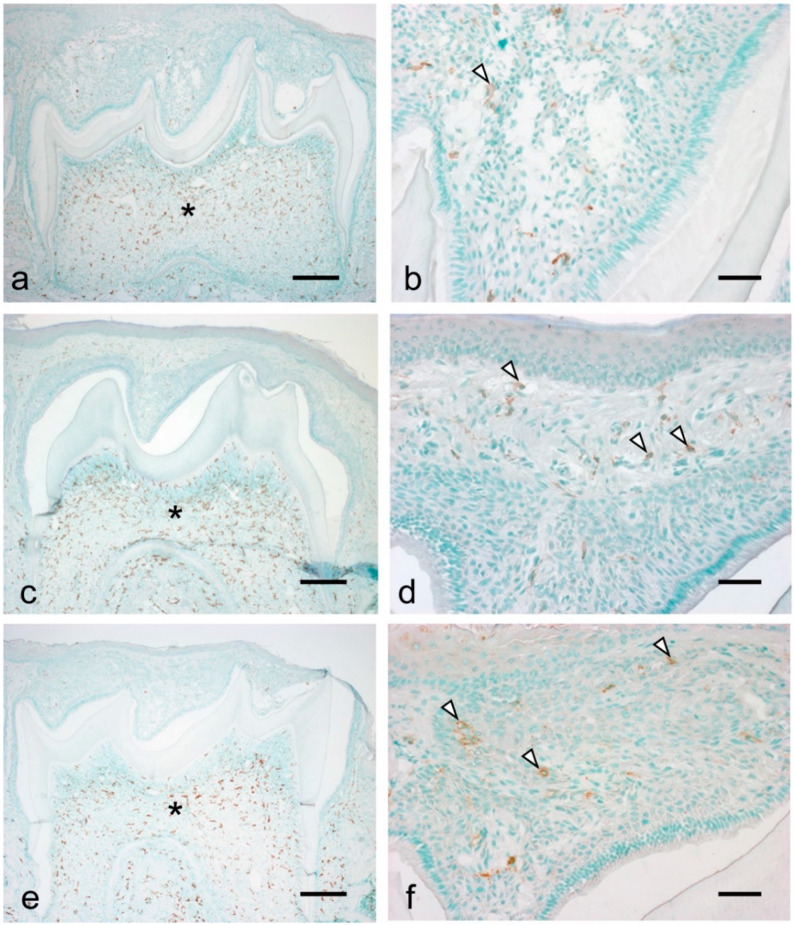
Localization of macrophages during the tooth eruption at 7 dPN (**a**,**b**), 14 dPN (**c**,**d**), and 19 dPN (**e**,**f**). Many residential macrophages were detected in the dental pulp in all stages (asterisks). From 14 dPN, macrophages were easily detected in the connective tissue overlying the tooth organ (**d**,**f**, arrowheads). Bars = 200 μm in (**a**,**c**,**e**); 50 μm in (**b**,**d**,**f**).

Considering the observed significant increase in inflammatory cytokines and chemokines during tooth eruption, we examined the migration of inflammatory cells, including macrophages and neutrophils, during the eruption. There were numerous resident macrophages in the dental papilla at all examined stages (Figure 4a,c,e, asterisks). Further, macrophages were detected in the tissue overlying the tooth organ at all stages (Figure 4b,d,f, arrowheads). At 7 dPN, the macrophages were localized to the enamel organ (Figure 4b). At 14 to 19 dPN, the macrophages were detected in the connective tissue overlying the tooth organ (Figure 4d,f).

**Figure 5:**
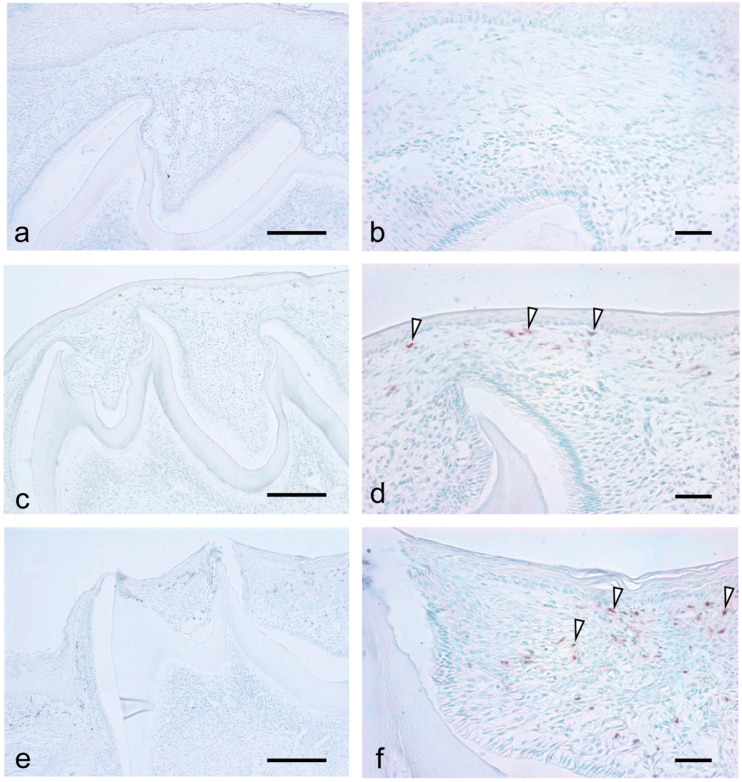
Localization of neutrophils during the tooth eruption at 7dPN (**a**,**b**), 14 dPN (**c**,**d**), and 19 dPN (**e**,**f**). No neutrophils were detected in the dental pulp in all stages. At 14 dPN, neutrophils were easily detected in the connective tissue overlying the tooth organ ((**d**), arrowheads), and the number of neutrophils were increased at 19 dPN ((**f**), arrowheads). Bars = 200 μm in (**a**,**c**,**e**); 50 μm in (**b**,**d**,**f**).

Neutrophils were not detected in the dental papilla at all examined stages (Figure 5a,c,e). At 7 dPN, there was no neutrophil infiltration in the enamel organ and the tissue overlying the enamel organ (Figure 5a). At 14 dPN, there were neutrophils in the tissue overlying the enamel organ (Figure 5d, arrowheads), and at 19 dPN, there was an increased number of infiltrating neutrophils (Figure 5f, arrowheads).

## 4. Discussion

The stratum intermedium changed to the papillary layer with the development of ameloblasts from the secretory stage to the maturation stage. Concomitantly with this structural change, papillary layer cells began to express ICAM-1. Our previous studies indicated that the origin of the junctional epithelium was the ICAM-1 expressing papillary layer [16,17,25]. ICAM-1 expression is regulated by proinflammatory cytokines, including IL-1 and TNF-alpha [26,27].

It has been indicated that tooth eruption requires the presence of a dental follicle (DF), alveolar bone resorption for an eruption pathway, and alveolar bone formation at the base of the bony crypt. Inflammatory cytokines such as IL-1α are increased in teeth with periapical lesions [28]. In this study, the expression of ICAM-1 in the papillary layer was after the alveolar bone covering the erupting tooth. Therefore, the induction of ICAM-1 was not link cytokine participating bone resorption.

In the present study, RT-PCR analysis revealed a significant increase in IL-1β expression in the covering tissue of the tooth organ during tooth eruption. Our findings suggest that proinflammatory cytokine expression induces ICAM-1 expression in the papillary layer. Moreover, blood vessels showed strong ICAM-1 expression within the covering tissue.

De Pizzol et al. [21] reported reduced microvascular density in the covering tissue during tooth eruption, which is indicative of severe hypoxia in this tissue. We observed significantly increased HIF-1α expression during tooth eruption. Moreover, HIF-1α expression regulates inflammation [23]. These results suggest that sequential molecular events occur during tooth eruption, where HIF-1α expression, through reduced microvasculature, induces IL-1β expression, which in turn induces ICAM-1 expression in the papillary layer cells and endothelial cells in the covering tissue.

Destruction of the covering tissue of the tooth organ is required for tooth eruption. Inflammation can induce tissue destruction. It has been recently shown that innate inflammation is involved in the onset of several diseases, including type 1 diabetes and Parkinson’s disease [29,30]. During tooth eruption, there were more apoptotic cells in the lamina propria covering the tooth organ [21]. Several danger signals by apoptosis coupled with hypoxia might induce the onset of innate inflammation during tooth eruption.

In this study, there was a mild inflammatory infiltrate predominated with neutrophils and macrophages in the covering tissue of the tooth organ during tooth eruption; de França Landim et al. [31] reported similar phenomena in rats. Further, RT-PCR results revealed a significant increase in the expression of MIP-2 and KC, which are the chemotactic factors of macrophages and neutrophils, respectively. Immunohistochemical analysis revealed KC and MIP-2 expression in the papillary layer and adjacent epithelial cells covering the tooth organ.

We have previously reported constitutive KC and MIP-2 expression in the junctional epithelium (JE), even in the germ-free condition for cell migration into JE [16]. These results indicate that the JE originates from papillary layer cells and that KC and MIP-2 expression in the JE might maintain the frontline barrier by neutrophil and macrophage migration to the JE without extrinsic stimulation.

MMP expression has been reported in the degradation of the lamina propria of the eruptive pathway of rat molar tooth [32,33]. Neutrophils and macrophages synthesize MMPs in response to proinflammatory cytokines [34,35]. These findings suggest that rapid degradation of the covering connective tissue over the tooth organ by neutrophils and macrophages might be a prerequisite for stable tooth eruption.

Eruption gingivitis is a common symptom in pediatric dental practice [36]. Eruptive gingivitis is known to be induced by oral bacterial revelation. Our findings suggest that noninfectious inflammation is already induced during tooth eruption and that the inflammation is augmented by the bacterial infection.

## 5. Conclusions

The tissue remodeling during the tooth eruption was regulated by the noninfectious inflammation induced from the onset of HIF-1α expression by the hypoxia of the tissue overlying the tooth organ. Our results indicated that the noninfectious inflammation might participate in not only the onset and/or the exacerbation of diseases but also the tissue development.
